# High-Sensitivity C-Reactive Protein Relationship with Metabolic Disorders and Cardiovascular Diseases Risk Factors

**DOI:** 10.3390/life11080742

**Published:** 2021-07-26

**Authors:** Małgorzata Koziarska-Rościszewska, Anna Gluba-Brzózka, Beata Franczyk, Jacek Rysz

**Affiliations:** Department of Nephrology, Hypertension and Family Medicine, Medical University of Lodz, Żeromskiego 113, 90-549 Łódź, Poland; malgorzata.koziarska-rosciszewska@umed.lodz.pl (M.K.-R.); beata.franczyk-skora@umed.lodz.pl (B.F.); jacek.rysz@umed.lodz.pl (J.R.)

**Keywords:** hsCRP, cardiovascular disease, metabolic syndrome

## Abstract

Background. Chronic inflammation is considered to be involved in the development of CVD. It is important to find a simple test that enables the identification of patients at risk and that may be used in primary care. The aim of this study is to investigate the associations of high-sensitivity C-reactive protein (hsCRP) with selected factors—age, gender, obesity, dyslipidemia, diabetes, hyperuricemia, vitamin D-25(OH)D, cardiovascular diseases (CVD), coronary heart disease, cerebrovascular disease, and hypertension. Results. Statistically significant correlations were found between hsCRP and the following: age (rs = 0.304, *p* = 0.0000); gender (female) (*p* = 0.0173); BMI (rs = 0.295, *p* = 0.0001); waist circumference (rs = 0.250, *p* = 0.0007); dyslipidemia (*p* = 0.0159); glycemia (rs = 0.173, *p* = 0.0207); and significant negative correlations between hsCRP and 25(OH)D (rs = −0.203, *p* = 0.0065). In patients with CVD, hypertension, diabetes, or visceral obesity, hsCRP was significantly higher than in the subgroup without these disorders. There was a statistically significant relationship between hsCRP and the number of the metabolic syndrome elements (*p* = 0.0053). Conclusions. The hsCRP test seem to be a simple test that may be used at the primary care level to identify patients at risk of metabolic disorders, CVD, and hypertension. Vitamin D concentration may be a determining factor of systemic inflammation (it may have a modulating effect).

## 1. Introduction

Chronic inflammation is considered to be involved in the development of cardiovascular diseases (CVD), such as coronary heart disease and cerebrovascular disease, and metabolic disorders [1,2,3,4]. Chronic low-grade inflammation has been also proposed to be a key factor for both metabolic syndrome (MetS) and subsequent clinical outcomes [5,6]. The presence of chronic low-intensity inflammation is also associated with the disruption of vascular endothelium glycocalyx by C-reactive protein (CRP), which leads to its dysfunction and increases the susceptibility to proatherogenic factors [4]. Inflammation-induced vascular changes cannot be evaluated with the use cardiac imaging methods; therefore, the biomarkers enabling the determination of such alterations are of high value [7,8,9]. Moreover, methods for the specific and sensitive identification of patients who are at risk of development of cardiovascular diseases are highly needed. Serum high-sensitivity CRP (hsCRP), which is a biomarker of inflammation, may meet these criteria. In healthy individuals, the concentration of CRP in blood does not usually exceed 10 mg/L; however, following stimulation, it can increase even over 1000 times [7,8,9]. hsCRP remains stable in samples for a long time, and its levels can be simply, rapidly, and cheaply determined [7,8]. The basal level of this protein depends on numerous factors, including the patient’s age, sex, race, ethnicity, hormonal status, obesity, smoking, alcohol consumption, diet, presence of infectious agent, disease duration, comorbidities, used drugs, and genetic polymorphisms [8]. Many prospective cohort studies have demonstrated the relationship between higher CRP levels and cardiovascular risk both in patients with established disease and in those at risk of atherosclerosis [10]. Therefore, it appears that hsCRP measurement can prove valuable in primary and secondary CVD prophylaxis [8]. Moreover, the level of this marker can be indicative of disease severity, treatment efficacy, and outcome prognosis [11]. On the other hand, multiple cohorts and meta-analyses show that hsCRP measurement may be comparable as a risk predictive factor with evaluation of HDL or total cholesterol, which are recommended in current European guidelines also related to primary prevention [12].

The results of the Physician’s Health Study indicated that the risk of stroke is twice as high, while the risk of myocardial infarction is three times higher in individuals with higher basal hsCRP concentration [13]. In turn, the Women’s Health Study demonstrated that hsCRP is a better prognostic factor for cardiovascular events compared to lipids or homocysteine [14]. Finally, Yoshinaga et al. [15] observed that elevated hsCRP is an important risk factor for in-hospital mortality among patients with CVD. High-sensitivity C-reactive protein has been also found to correlate with metabolic syndrome in many studies [16,17,18].

The aim of our study was to investigate the relationship between hsCRP and selected factors, such as age, gender, obesity, dyslipidemia, diabetes, hyperuricemia, vitamin D serum concentration (25(OH)D), CVD, and hypertension.

## 2. Materials and Methods

The study group consisted of 180 primary care patients (121 women and 59 men) undergoing laboratory tests for any reason. All of them had anthropometric measurements and laboratory tests (hsCRP, lipid profile, glycemia, uricemia, and 25(OH)D) performed with the use of the COBAS INTEGRA 400 plus analyzer. LDL was calculated with Friedewald formula. Information on chronic diseases was collected. The research protocol was approved by the Ethics Committee of Medical University of Lodz, Poland and complies with the Declaration of Helsinki. Informed consent was obtained from all patients before the enrolment into study.

### Statistical Analysis

The obtained data were statistically analyzed using STATISTICA, v.10. StatSoft, Inc., Tulsa, OK, USA (2011). A *p*-value ≤ 0.05 was considered as significant in all tests. Conformity of distribution of quantitative variables with normal distribution was analyzed with the use of the Shapiro–Wilk W test. The Mann–Whitney U test was used to compare differences between two independent groups when the dependent variable was either ordinal or continuous, and the Kruskal–Wallis one-way test was used for the analysis of variance, while the z test and Spearman correlation were used for multiple comparisons.

## 3. Results

The study group consisted of 121 women (67.2%) and 59 men (32.8%). The mean age of the participants was 60.8 ± 17.0. BMI ≥ 25.0 was observed in 69.4% of the participants and abnormal waist circumference (women > 80 cm, men > 94 cm) in 63.3% of the subjects.

Nearly four out of five subjects (80.6%) had dyslipidemia. Elevated total cholesterol (≥200 mg/dL), LDL (≥100 mg/dL), and triglycerides (≥150 mg/dL) were reported in 43.5%, 59.4%, and 22.4% of the participants, respectively, while decreased HDL cholesterol levels (men < 40 mg/dL, women < 50 mg/dL) were observed in 19.4% of the individuals.

The concentration of hsCRP ≥ 0.5 mg/L was found in 11.7% of the participants. Approximately 1/3 of the study group individuals (34.3%) had abnormal serum glucose (≥100 mg/dL). A total of 17.2% of the patients had elevated uric acid (>7 mg/dL), and 83.7% had a low concentration (<30 ng/mL) of vitamin D.

Diseases of the circulatory system were found in 62.2% of the subjects, while 74.4% had hypertension. Nearly one-fourth of the subjects (23.0%) had diabetes. Visceral obesity was observed in 71.7% of the examined individuals.

Selected descriptive statistics for the studied group are summarized in Table 1.

### 3.1. The Relationship between hsCRP Level and Age, Gender, and Selected Anthropometric and Laboratory Parameters

We analyzed the relationship between hsCRP concentration and the age and gender of the study participants. There was a statistically significant positive correlation between the concentration of this factor and the age of the subjects (rs = 0.304, *p* < 0.00001, Table 2). The concentration of hsCRP increased with advancing age. There was also a significant relationship between the concentration of hsCRP and the gender of patients in the study group. Median hsCRP concentration was higher in the subgroup of women than the subgroup of men (*p* = 0.0173, Table 3).

We also assessed the relationship between hsCRP concentration and selected anthropometric parameters, including BMI and waist circumference. In the study group, the hsCRP level significantly correlated with BMI values (rs = 0.295, *p* = 0.0001, Table 2); the concentration of hsCRP increased with the increase in BMI. Moreover, we compared subgroups of patients with a normal BMI (BMI < 25), those overweight (30 ≥ BMI ≥ 25), and obese (BMI > 30) individuals. We observed marked differences between these subgroups (*p* = 0.0010, Table 3). Subsequent analysis revealed that the concentration of hsCRP was significantly lower among people with normal BMI compared to obese individuals (*p* = 0.0007, Table 3). In our study group, the waist circumference significantly correlated with the concentration of hsCRP (rs = 0.250, *p* = 0.0007, Table 2). The level of hsCRP increased with increasing waist circumference. Moreover, median hsCRP concentration was significantly higher in the subgroup of individuals with a high waist circumference (M > 94 cm, K > 80 cm) compared to the subgroup of people with a normal waist circumference (*p* = 0.0008, Table 3).

We also examined whether the serum level of hsCRP correlates with lipid profile: total cholesterol, LDL cholesterol, HDL cholesterol, and triglycerides. There were no significant correlations between the hsCRP concentration and the above mentioned parameters in the study group (Table 2). Concentrations of hsCRP also did not differ significantly between the subgroups of patients with normal and elevated concentrations of these lipoproteins (Table 3). Interestingly, there was a statistically significant relationship between the concentration of hsCRP and the occurrence of dyslipidemia. In a subgroup of patients with dyslipidemia, median hsCRP concentration was significantly higher than in the subgroup without lipid disorders (*p* = 0.0159, Table 3).

Finally, we searched for correlations between hsCRP levels and glucose, uric acid, and vitamin D concentration (25(OH)D) in the study group. The concentration of hsCRP significantly positively correlated with the glucose concentration (rs = 0.173, *p* = 0.0207; Table 2) and significantly negatively with 25(OH)D (rs = -0.203, *p* = 0.0065; Table 2). With the increase in hsCRP concentration, there was an increase in glucose concentration and a decrease in 25(OH)D. However, no significant correlation was found between hsCRP and uric acid (rs = 0.090, *p* = 0.2300, Table 2).

### 3.2. The Relationship between the Concentration of Hscrp and the Occurrence of Selected Diseases, the Number of Elements of the Metabolic Syndrome, and the Number of Risk Factors for Cardiovascular Disease Was Observed

We analyzed possible relationships between the concentration of hsCRP and the occurrence of the following: CVD, hypertension, diabetes, and visceral obesity. In the subgroup of the patients suffering from any of the analyzed diseases, the concentration of hsCRP was significantly higher compared to that in the subgroup of subjects without this disorder (Table 4).

**Figure 1:**
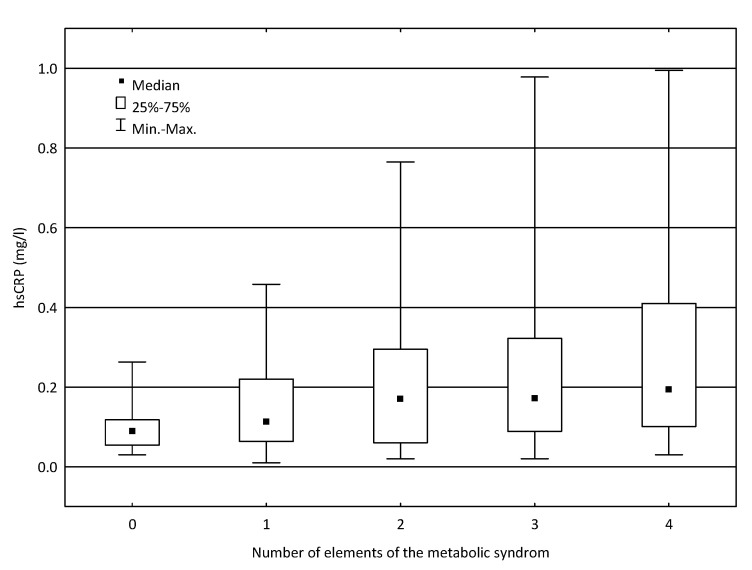
Relationship between the concentration of hsCRP and the number of elements of the metabolic syndrome.

Moreover, we examined possible relationships between the concentration of hsCRP and the number of elements of the metabolic syndrome (waist circumference M > 94 cm in men or >80 cm in women, glucose concentration> 99 mg/dL, current dyslipidemia, and current hypertension). There was a statistically significant association between the hsCRP concentration and the number of these factors (*p* = 0.0053, Figure 1, Table 5). The comparison of hsCRP levels in subgroups of patients with different number of elements of the metabolic syndrome revealed considerable differences between the group without any component of the syndrome and the group of patients with three elements of the syndrome (*p* = 0.0206) and between patients without any component of the syndrome and the group of patients with four elements of the syndrome (*p* = 0.0036, Table 5).

An analogous analysis was performed for the relationship between hsCRP and the number of risk factors for CVD (male gender, current obesity, current hypertension, triglyceride concentration ≥150 mg/dL, HDL cholesterol <40 mg/dL in men or <50 mg/dL in women, and glucose concentration > 99 mg/dL). There was no statistically significant relationship between the concentration of hsCRP and the number of these factors (*p* = 0.1451, Table 5).

## 4. Discussion

In this study we assessed the relationship between hsCRP levels and patients’ characteristics. We observed that hsCRP concentration strongly correlated with the age and gender of the patients. Levels of hsCRP were higher in older patients. Similar observations were made by Kawamoto et al. [19], as well as by Wang et al. [20] in the Chinese population. Moreover, the study of the effect of age on markers of inflammation demonstrated that age positively correlated with CRP levels [21]. Moreover, on the basis of a 10-year follow-up of the Framingham Offspring Study, McCabe et al. [22] suggested that CRP level could serve an additional parameter for aging assessment, which could improve the performance of the healthy ageing index (HAI) in recognizing the healthiest older adults.

Our study also revealed higher hsCRP in the subgroup of women than in the subgroup of men (*p* = 0.0173). This result is in agreement with the result of Premanath et al. [23], who found higher levels in women (both obese and with normal weight) compared to men. Wener et al. [24], who evaluated the differences in the upper normal limit of CRP in women and men, suggested that demographic data, including age, sex and race, should be utilized to adjust the upper reference limit for CRP. In their opinion, future studies involving hsCRP ought to be based on sex-specific analyses due to significant differences in CRP levels between the genders. In turn, McConnell et al. [25] found that differences in hsCRP between genders were independent of age differences.

We also analyzed the relationship between hsCRP concentration and selected anthropometric parameters (BMI and waist circumference). In the study group, its concentration significantly correlated with BMI value; the concentration of hsCRP increased with the increase in BMI. Moreover, hsCRP concentration also markedly differed between subgroups of patients, namely, those with a normal BMI (BMI < 25), overweight participants (30 ≥ BMI ≥ 25), and obese participants (BMI > 30). We demonstrated that the level of hsCRP was significantly lower among individuals with normal BMI compared to obese individuals. This is in agreement with another Polish study that revealed that abdominal obesity and BMI ≥ 30 kg/m^2^ are factors increasing the probability of elevated inflammatory activity [26]. In turn, Japanese scientists examining the impact of body anthropometric parameters and serum hsCRP on HOMA-IR found that concomitant obesity and elevated systemic inflammation might synergistically contribute to increased insulin resistance [27]. Jeemon et al. [28] also revealed that clinical measurements of adiposity, such as BMI and abdominal obesity, correlated with the systemic inflammatory state of individuals. An observational cohort study revealed that hsCRP was significantly correlated with BMI [29]. Moreover, they indicated that the hsCRP/BMI ratio was independently and positively associated with the occurrence of major adverse cardiovascular events (MACE). In their opinion, patients with ACS with high hsCRP plus overweight had the same risk of MACE as those with lower hsCRP but normal weight. Therefore, they stated that the C-reactive protein level should be adjusted by BMI to reflect the prognosis of patients with ACS. Furthermore, Chinese research observed that hsCRP correlated with BMI as well as with most of the known CVD risk factors [20]. Interesting findings come from an Australian study demonstrating that hsCRP and obesity are associated with elevated blood pressure in young females [30].

In our study group, waist circumference significantly correlated with the concentration of hsCRP. The concentration of hsCRP increased with increasing waist circumference. Obesity is a well-known factor associated with a high risk of insulin resistance (IR) and its complications. Taiwanese research observed that central fat distribution of adipose tissue correlated with increased risk of IR and chronic inflammation. Out of five inflammatory markers (adiponectin, leptin, tumor necrosis factor-α TNF-α, resistin, and hsCRP), variances in hsCRP and adiponectin levels could be explained by intraperitoneal fat [31]. Moreover, a study on an Indian industrial population revealed that BMI and abdominal obesity correlated with the systemic inflammatory state of individuals [28].

In our study, we failed to identify an association between lipid profile and hsCRP level. The concentration of hsCRP did not differ significantly between the subgroups of patients with normal and elevated concentrations of the lipid parameters. Interestingly, there was a statistically significant relationship between the concentration of hsCRP and the occurrence of dyslipidemia. In a subgroup of patients with dyslipidemia, median hsCRP concentration was significantly higher compared to the subgroup of subjects without lipid disorders (*p* = 0.0159). In contrast, Kawamoto et al. [19] found that lipid disorders, especially triglycerides and HDL, were significantly associated with hsCRP. In turn, in Chinese research, elevated hsCRP (>1.80 mg/L) positively correlated with LDL and negatively with HDL/total cholesterol, LDL/total cholesterol, and total cholesterol independently [20]. These discrepancies may be the result of different study groups. However, the limitation of our study was the lack of information of current statin therapy in the examined patients.

In our study, we demonstrated that the concentration of hsCRP significantly positively correlated with glucose concentration and considerably negatively with vitamin D concentration. With the increase in hsCRP concentration, there was an increase in glucose concentration and a decrease in vitamin D concentration. No significant correlation was found between hsCRP and uric acid concentrations. Similar results were obtained by Leiva et al. [32], who revealed that hsCRP was significantly associated with glycemia levels (*p* = 0.009). However, they also indicated the relationship between hsCRP and uric acid (*p* = 0.001), which was noted in our study. Their ROC curves analysis demonstrated that a uric acid level of 3.9 mg/dL was a cut-off point to predict a high value of hsCRP. Individuals with uric acid values exceeding 3.9 mg/dL and normal glycemia had 3.5-fold higher risk of having hsCRP levels over 3.0 mg/L [32]. Uric acid is considered to be an important element of metabolic processes and contributes to CVD. Results of the MONICA/KORA cohort study confirmed that high uric acid levels were independently associated with CVD mortality as well as all-cause mortality in middle-aged men from the general population in Germany [33]. Chinese scientists found that uric acid was independently associated with hsCRP in diabetic patients, which indicates the presence of chronic inflammation in patients with hyperuricemia [34]. Kawamoto et al. [19] also confirmed significant association of hsCRP with uric acid in men and women from a single Japanese community. The differences with the results of our study may arise from some population dissimilarities. Moreover, a Finnish study—METSIM—revealed that hsCRP levels were associated with adverse changes in insulin sensitivity and obesity-related traits, as well as with total mortality. Furthermore, hsCRP predicted changes in insulin sensitivity [35]. Interesting results come from the study conducted by Bagherniya et al.—hsCRP concentrations independently predict the development of diabetes, metabolic syndrome, and cardiovascular disease [36]. Moreover, Chinese research revealed that elevated hsCRP (>1.80 mg/L) was associated with most of the known CVD risk factors, including hyperglycemia [20]. The results of an Iranian study indicated that fasting blood glucose had the greatest association with hsCRP concentration [37]. Some studies found an inverse relationship between 25(OH)D and markers of inflammation, such as hsCRP. The question is whether vitamin D diminishes inflammation or whether inflammation reduces 25(OH)D levels [38]. The results of the National Health and Nutrition Examination Survey (NHANES) 2001–2006 show that in subjects with a 25(OH)D <53 nmol/L, serum 25(OH)D was inversely associated with CRP [39].

In our study, hsCRP levels were also significantly higher in subgroup of the patients suffering from CVD, hypertension, diabetes, and visceral obesity compared to subgroup of subjects without such disorders. These results are consistent with the results of other studies. The PREVEND Study, a prospective population-based cohort study in the Netherlands (including participants with and without metabolic syndrome), revealed that high hsCRP was independently associated with new-onset CVD and chronic kidney disease [40]. The results of the Women’s Ischemia Syndrome Evaluation (WISE) study also confirmed a significant, positive correlation among BMI, blood pressure, and levels of inflammatory factors [41]. Moreover, Krzesiński et al. [26], examining patients with hypertension, revealed that the increased level of inflammatory markers, especially hsCRP, correlated with complex metabolic disturbances and that CV risk increased when the inflammatory markers levels (especially hsCRP) were elevated. Interestingly, our observations indicate that in non-obese patients with hypertension, hsCRP was higher than in non-obese patients without hypertension. In our study, we also found a significant relationship between the concentration of hsCRP and the number of elements of the metabolic syndrome (waist circumference M > 94 cm in men or > 80 cm in women, glucose concentration > 99 mg/dL, current dyslipidemia, and current hypertension). We observed higher hsCRP in the group of patients with three elements of the syndrome (*p* = 0.0206) and the group of patients with four elements of the syndrome (*p* = 0.00360) when compared to the group without any component of the syndrome. Similar results were obtained by Mirhafez et al. [37]. In their study, the concentration of serum hsCRP increased progressively with the number of metabolic syndrome components; moreover, fasting blood glucose had the greatest association with hsCRP concentration. A relationship between metabolic syndrome and high CRP was also found in a Chilean study, but it was confirmed only in men. The same association was observed for high triglyceride levels and high waist circumference. In women, the only relationship observed was between abdominal obesity and very high CRP [42].

## 5. Conclusions

hsCRP analysis seems to be a simple test that may be used at the primary care level to identify patients at risk of metabolic disorders (obesity, dyslipidemia, and diabetes), CVD, and hypertension. Patients with elevated hsCRP should undergo screening for the presence of other CVD risk factors. Vitamin D serum concentration may be a factor influencing the processes of systemic inflammation.

## Figures and Tables

**Table 1:** Selected descriptive characteristics for the study group.

Parameter	N	Mean	SD	Median	Min.	Max.	IQR	CV
Age (years)	180	60.8	17.0	61.5	23.0	95.0	22.5	27.9
Weight (kg)	180	77.4	16.7	74.9	41.5	150.0	20.0	21.5
Height (cm)	180	166.1	9.78	165.0	145.0	193.0	13.50	5.89
BMI	180	28.0	5.4	27.5	16.6	49.5	6.7	19.4
Waist circumference (cm)	180	91.6	13.00	90.5	60.0	141.0	16.50	14.18
hsCRP (mg/L)	180	0.223	0.202	0.159	0.010	0.995	0.214	90.587
Total cholesterol (mg/dL)	170	196.4	43.9	190.5	107.0	347.0	61.0	22.3
LDL cholesterol (mg/dL)	170	111.3	38.4	108.5	29.0	244.0	52.0	34.5
HDL cholesterol (mg/dL)	170	60.5	16.7	59.0	28.0	108.0	26.0	27.5
Triglycerides (mg/dL)	170	120.7	50.9	105.5	46.0	267.0	63.0	42.1
Glucose (mg/dL)	178	98.0	19.6	93.5	61.0	225.0	17.0	19.9
Uric acid (mg/dL)	180	5.6	1.4	5.5	1.7	8.8	2.5	25.8
Vitamin D (ng/mL)	178	23.5	9.4	23.0	8.0	67.0	10.0	39.8

**Table 2:** Relationship between hsCRP concentration and age and selected anthropometric and laboratory parameters.

Parameter	rs *	*p* Value
Age (years)	0.304	0.0000
BMI	0.295	0.0001
Waist circumference (cm)	0.250	0.0007
Total cholesterol (mg/dL)	−0.119	0.1226
LDL cholesterol (mg/dL)	−0.149	0.0521
HDL cholesterol (mg/dL)	0.002	0.9805
Triglycerides (mg/dL)	−0.084	0.2770
Glucose (mg/dL)	0.173	0.0207
Uric acid (mg/dL)	0.090	0.2300
Vitamin D (ng/mL)	−0.203	0.0065

* Spearman’s rank correlation coefficient.

**Table 3:** Relationship between hsCRP concentration and gender and selected anthropometric and laboratory parameters.

Parameter		hsCRP (mg/L)					*p* Value
		N	Median	Min.	Max.	IQR	
Gender	women	121	0.172	0.010	0.995	0.224	0.0173 *
	men	59	0.107	0.020	0.978	0.143	
BMI	<25	55	0.103	0.010	0.818	0.156	0.0010 ** 0.0843 ^#^ 0.0007 ^##^ 0.2245 ^###^
	<25;30>	73	0.159	0.020	0.995	0.166	
	>30	52	0.203	0.036	0.767	0.281	
Waist circumference (cm)	M > 94, K > 80	114	0.183	0.030	0.995	0.241	0.0008
	M ≤ 94, K ≤ 80	66	0.109	0.010	0.818	0.150	
Total cholesterol (mg/dL)	≥200	74	0.132	0.020	0.995	0.185	0.1290 *
	<200	96	0.164	0.010	0.978	0.232	
LDL cholesterol (mg/dL)	≥100	101	0.123	0.020	0.995	0.185	0.0849 *
	<100	69	0.181	0.010	0.978	0.230	
HDL cholesterol (mg/dL)	M < 40, K < 50	33	0.175	0.020	0.765	0.279	0.0602 *
	M ≥ 40, K ≥ 50	137	0.124	0.010	0.995	0.192	
Triglycerides (mg/dL)	≥150	38	0.144	0.020	0.978	0.189	0.6849 *
	<150	132	0.156	0.010	0.995	0.216	
Dyslipidemia	present	145	0.172	0.020	0.995	0.229	0.0159 *
	absent	35	0.107	0.010	0.636	0.149	
Glucose (mg/dL)	>99	61	0.181	0.020	0.995	0.314	0.0889 *
	≤99	117	0.147	0.010	0.978	0.180	
Uric acid (mg/dL)	>7	31	0.180	0.032	0.700	0.301	0.5815 *
	≤7	149	0.159	0.010	0.995	0.198	
Vitamin D (ng/mL)	<30	149	0.170	0.010	0.995	0.236	0.0870 *
	≥30	29	0.109	0.020	0.450	0.119	

* Mann–Whitney U test; ** Kruskal–Wallis one-way analysis of variance. ^#^ BMI < 25 vs. BMI <25;30>, ^##^ BMI < 25 vs. BMI > 30, ^###^ BMI <25;30> vs. BMI > 30; z test for multiple comparisons.

**Table 4:** Relationship between hsCRP concentration and the occurrence of selected diseases.

Parameter		N	Median	Min.	Max.	IQR	*p* Value
Cardiovascular disease	present	112	0.178	0.020	0.995	0.270	0.0023 *
	absent	68	0.113	0.010	0.765	0.156	
Hypertension	present	134	0.177	0.010	0.995	0.274	0.0018 *
	absent	46	0.106	0.030	0.708	0.133	
Diabetes	present	41	0.236	0.020	0.818	0.299	0.0270 *
	absent	137	0.147	0.010	0.995	0.175	
Visceral obesity	present	129	0.180	0.010	0.995	0.249	0.0023 *
	absent	51	0.110	0.020	0.818	0.143	

* Mann–Whitney U test.

**Table 5:** Relationship between hsCRP concentration and the number of elements of the metabolic syndrome and the number of risk factors for cardiovascular disease.

Parameter		N	Median	Min.	Max.	IQR	*p* Value
Number of elements of the metabolic syndrome	0	18	0.089	0.030	0.263	0.064	0.0053 **
	1	20	0.113	0.010	0.458	0.156	
	2	33	0.170	0.020	0.765	0.235	0.0206 ^#^
	3	63	0.171	0.020	0.978	0.233	0.0036 ^##^
	4	44	0.194	0.030	0.995	0.309	>0.050 ^###^
Number of risk factors of cardiovascular disease	0	19	0.097	0.033	0.449	0.114	0.1451 **
	1	36	0.137	0.010	0.708	0.167	
	2	50	0.176	0.022	0.995	0.264	
	3	39	0.175	0.032	0.978	0.221	
	4	19	0.170	0.020	0.767	0.261	
	5	6	0.261	0.020	0.700	0.371	

** Kruskal–Wallis one-way analysis of variance. ^#^ 0 vs. 3, ^##^ 0 vs. 4, ^###^ other comparisons; z test for multiple comparison.

## Data Availability

All data used in this study was included in this manuscript. There are no other sources of additional data.
